# Supplementary material for: Decellularized vascularized bone grafts as therapeutic solution for bone reconstruction: A mechanical evaluation
Source: PLoS One. 2023 Jan 13;18(1):e0280193. doi: 10.1371/journal.pone.0280193 (PMC9838862; doi:10.1371/journal.pone.0280193)
Supplement: S1 Table — Table showing all the results obtained during our pull-out tests. (DOCX) [file pone.0280193.s001.docx]

|  | Central Screw | | Lateral Screw | | Medial Screw | | All Screws | |
| --- | --- | --- | --- | --- | --- | --- | --- | --- |
| **NATIVE VS DECELLULARIZED PROTOCOL 1** | | | | | | | | |
|  | Native | Protocol 1 | Native | Protocol 1 | Native | Protocol 1 | Native | Protocol 1 |
| Number of Datas | 6 | 6 | 6 | 6 | 6 | 6 | 18 | 18 |
|  | Maximum Force (N) | | | | | | | |
| Min – Max | 70 – 235.2 | 100.6 – 180.4 | 94.4 – 272.3 | 138 – 205 | 128 – 358.4 | 213.7 - 361 | 70 – 358.4 | 100.6 - 361 |
| Mean (Sd) | 140.6 (54.1) | 146.9 (29.0) | 201.0 (69.4) | 172.1 (27.3) | 251.2 (78.8) | 265.4 (63.5) | 197.6 (79.2) | 194.8 (66.4) |
| Relative change% (Raw value (N)) | -4.5% (-6.3) | | +14.4% (28.9) | | -5.7% (-14.2) | | +1.4% (2.8) | |
|  | Transmitted Energy (mJ) | | | | | | | |
| Min — Max | 36.9 – 223.5 | 73.8 – 192.4 | 71.12 – 360.5 | 112 — 309.9 | 60.5 – 589.4 | 79.4 – 194 | 36.9 – 589.4 | 73.8 – 309.9 |
| Mean (Sd) | 109.4 (75.7) | 124 (42.2) | 207.5 (106.9) | 185.7 (96.2) | 255.2 (198.2) | 125.5 (41.8) | 190.7 (143.2) | 145.1 (68.1) |
| Relative change% (Raw value (J)) | +30.6% (33.45) | | +5.1% (10.7) | | +61.3% (156.4) | | 39.4% (75.1) | |
| **DECELLULARIZED PROTOCOL 1 VS PROTOCOL 2** | | | | | | | | |
|  | Protocol 1 | Protocol 2 | Protocol 1 | Protocol 2 | Protocol 1 | Protocol 2 | Protocol 1 | Protocol 2 |
| Number of Datas | 6 | 7 | 6 | 7 | 6 | 7 | 18 | 21 |
|  | Maximum Force (N) | | | | | | | |
| Min – Max | 100.6 – 180.4 | 52.9 – 110.8 | 138 – 205 | 64.9 – 137.1 | 213.7 - 361 | 114.9 – 237.7 | 100.6 - 361 | 52.9 – 237.7 |
| Mean (Sd) | 146.9 (29.0) | 72.2 (20.7) | 172.1 (27.2) | 105.2 (30.0) | 265.4 (63.5) | 174.2 (43.7) | 194.8 (66.4) | 117.2 (53.1) |
| Difference of Mean | +49.8% (74.7) | | +38.9% (66.9) | | +34.3% (91.16) | | +39.8% (77.6) | |
|  | Transmitted Energy (${10}^{-3}$J) | | | | | | | |
| Min — Max | 73.8 – 192.4 | 25.06 – 92.1 | 112 — 309.9 | 60.9 – 162.7 | 79.4 – 194 | 96.4 – 289.1 | 73.8 – 309.9 | 25.1 - 289.1 |
| Mean (Sd) | 124 (42.2) | 57.5 (27.6) | 185.7 (96.2) | 98.4 (35.7) | 125.5 (41.8) | 176.1 (65.9) | 145.1 (68.1) | 110.7 (66.7) |
| Difference of Mean | +53.6% (66.5) | | +47% (87.3) | | -40.3% (-50.6) | | 23.4% (34.4) | |

**S1 Table: Full results of the pull-out test.** Table showing all the results obtained during our pull-out tests.
