# Supplementary material for: Decellularized vascularized bone grafts as therapeutic solution for bone reconstruction: A mechanical evaluation
Source: PLoS One. 2023 Jan 13;18(1):e0280193. doi: 10.1371/journal.pone.0280193 (PMC9838862; doi:10.1371/journal.pone.0280193)
Supplement: S2 Table — Table showing all the results obtained during our compression tests. (DOCX) [file pone.0280193.s002.docx]

|  | Native | D1 | | D2 |
| --- | --- | --- | --- | --- |
| Number of Datas | 6 | 6 | | 7 |
|  | Apparent elastic modulus (MPa) | | | |
| Minimum - Maximum | [482.7 – 695.7] | [468.2 – 919.4] | | [232.1 – 1075.5] |
| Mean (SD) | 570.8 (84.8) | 656.9 (180) | | 643.4 (312.9) |
| Difference of Mean | -14.9% (-85.1MPa) | | +2% (13.5MPa) | |

**S2 Table : Results of compression tests.** Table showing all the results obtained during our compression tests
