# Supplementary material for: Decellularized vascularized bone grafts as therapeutic solution for bone reconstruction: A mechanical evaluation
Source: PLoS One. 2023 Jan 13;18(1):e0280193. doi: 10.1371/journal.pone.0280193 (PMC9838862; doi:10.1371/journal.pone.0280193)
Supplement: S3 Table — Table showing all the results obtained during our 3 points bending tests. (DOCX) [file pone.0280193.s003.docx]

|  | Native | Decellularized – Protocol 1 | | Decellularized – Protocol 2 |
| --- | --- | --- | --- | --- |
| Number of Datas | 6 | 6 | | 7 |
|  | Maximum Force (N) | | | |
| Minimum - Maximum | [619.2 – 965.2] | [629.2 – 1196] | | [263.4 – 732] |
| Mean (SD) | 788.1 (125.3) | 833.1 (210.4) | | 479.4 (190.2) |
| Difference of Mean | -5.7% (-45N) | | +44.9% (353.7N) | |
|  | Bending Work ($\times10^{3}$J) | | | |
| Minimum - Maximum | [1.929 – 3.823] | [1.728 — 3.817] | | [0.373 – 2.215] |
| Mean (SD) | 2.8 (0.67) | 2.63 (0.8) | | 1.2 (0.69) |
| Difference of Mean | +6.2% (0.173) | | 53.1% (1.398) | |

**S3 Table: Results of 3 points Bending tests.** Table showing all the results obtained during our 3 points bending tests
