# Supplementary material for: Decellularized vascularized bone grafts as therapeutic solution for bone reconstruction: A mechanical evaluation
Source: PLoS One. 2023 Jan 13;18(1):e0280193. doi: 10.1371/journal.pone.0280193 (PMC9838862; doi:10.1371/journal.pone.0280193)
Supplement: S4 Table — Table showing all the results obtained during our indentation tests. (DOCX) [file pone.0280193.s004.docx]

|  | Native | Decellularized – Protocol 1 | | Decellularized – Protocol 2 |
| --- | --- | --- | --- | --- |
| Number of datas | 132 | 152 | | 209 |
|  | Hardness (HV) | | | |
| Min-Max | [0.5236 – 8.198] | [0.4784 – 9.352] | | [0.5731 – 12.55] |
| Mean (SD) | 3.44 (2.05) | 3.81 (2.19) | | 4.1 (2.91) |
| Difference of mean | -10.8% (-0.37) | | -7.6% (0.29) | |

**S4 Table: Results of the indentation tests.** Table showing all the results obtained during our indentation tests
