# Supplementary material for: Decellularized vascularized bone grafts as therapeutic solution for bone reconstruction: A mechanical evaluation
Source: PLoS One. 2023 Jan 13;18(1):e0280193. doi: 10.1371/journal.pone.0280193 (PMC9838862; doi:10.1371/journal.pone.0280193)
Supplement: Attachment — Submitted filename: Response to Reviewers.docx [file pone.0280193.s005.docx]

Paris, the 01/09/2022

Dear Editor, Dear Reviewers,

We thank you for having considered our paper entitled: Decellularized vascularized bone grafts as therapeutic solution for bone reconstruction: A mechanical evaluation.

We would like to sincerely thank you for your relevant comments and questions that will significantly help us to enhance the quality of the article.

In the following section, we respond to specific comments from reviewers. We also pointed in the article the main changes, that we colored according to one who induced it: Academic editor, Reviewer #1, Reviewer #2.

We thank you for considering another time this reviewed manuscript.

Academic editor,

1. Please ensure that your manuscript meets PLOS ONE's style requirements, including those for file naming.

We tried to ensure that our manuscript was as close as possible to the recommendations made by PLOS ONE. The article should now meet PLOS ONE's style requirements.

2. To comply with PLOS ONE submissions requirements, in your Methods section, please provide additional information regarding the experiments involving animals and ensure you have included details on (1) methods of sacrifice, (2) methods of anesthesia and/or analgesia, and (3) efforts to alleviate suffering.

Thank you for pointing out this lack. We have added this to our manuscript

3. Thank you for stating the following in the Acknowledgments Section of your manuscript:

"The authors would like to thank the Fondation des Gueules Cassées for its help and support for this study." We note that you have provided funding information that is not currently declared in your Funding Statement. However, funding information should not appear in the Acknowledgments section or other areas of your manuscript. We will only publish funding information present in the Funding Statement section of the online submission form. 
Please remove any funding-related text from the manuscript and let us know how you would like to update your Funding Statement. Currently, your Funding Statement reads as follows:

"This work was supported by Fondation des Gueules Cassées (Application N°41-2020, France) granted by Dr. UH
The funders had no role in study design, data collection and analysis, decision to publish, or preparation of the manuscript."

We apologize for this confusion. We have removed this part in Acknowledgments. The Funding Statement complete, now, your recommendations

4. Please include captions for your Supporting Information files at the end of your manuscript, and update any in-text citations to match accordingly. Please see our Supporting Information guidelines for more information: [http://journals.plos.org/plosone/s/supporting-information](http://journals.plos.org/plosone/s/supporting-information)

This was modified and added in the manuscript.

5. Quantitative results should be added to the abstract.

We added quantitative results in the manuscript.

6. Using 42 references in the introduction makes the novelty unclear. It is better that the innovation is highlighted in the introduction, compared to the literature review.

We have reduced the number of references in the introduction and try to express the novelty of our work by pointing out that this is the first time that this kind of mechanical comparison is performed. We also have tried to highlight the novelty of our paper in the introduction.

7. All formulations need references.

Added in the manuscript.

8. The discussion is poor. Obtained results must be compared to other results of other similar publications.

Thank you for your comment. We have added comparisons with the literature to enrich our discussion, but as it is the first time this kind of comparison is done the literature is poor on the subject.

9. The scale bar should be added to Figures 2, 4, 5, 6, and 7.

Done.

10. It is better to add a table to introduce the samples. What was the repeatability of testing? The number of specimens could be added to this new table.

Thank you for this relevant comment. We added a table to make sample allocations and repeatability easier for the reader to understand.

**Reviewer #1:**

1. Both protocols were completed by rinsing with PBS. It neutralizes the reaction of previously used detergents; however, the protocol should be completed by rinsing with deionized or distilled water to minimize the presence of detergent microelements in the graft. Maybe you used it but didn't mention it. Please add or explain if you don't use it how you confirm the complete release of detergent elements from the graft.

Thank you for bringing this to our attention. Indeed, we did wash with deionized water between each stage. We have added this point to the text (line 163 and line 170).

2. There are no legends (explanation) on figures and tables, what makes them difficult to understand.

We have added legends to the figures and tables to make them more understandable to the reader.

3. Why are the figure titles in the middle of the text and not next to the figures or separated?
We have tried to follow the publisher's recommendations. The figures are on a separate file and the titles and legends are in the manuscript at the request of the editor. We have uploaded a version of the manuscript with images within it for better readability.

4. What does it mean on line 206 "a) Pull-out Screw Test (Fig 4)" I did not understand why it is written here. I think it is not necessary to use A and B as a test, just name it simply.

Done.

5. On line 339 (in the results) the abbreviation GLMM appears. I think you mean "General Linear Mixed Model", but it is not explained in the text. Please explain all abbreviations.

Thank you for pointing that out. We corrected it in the text.

**Reviewer #2:**

1. After bone grafting at the site of bone defect, the biomechanical properties of the implant may be altered due to the in vivo biological environment. And this of the biomechanical performance of the decellularized sample in the organism precisely determines whether the defect site has a good osseointegration effect or not. Therefore, it is suggested that the authors may consider the inclusion of in vivo experiments.

Dear reviewer, thank you for this pertinent comment. We are currently planning an in vivo study on bone grafts decellularized by vascular injection. This study will be conducted over the coming year and will be the subject of an article on the histological, mechanical, and biological results.

2. In this article, Sodium Dodecyl Sulfate (SDS) and NaOH and H2O2 substitute based on the perfusion-decellularisation technique were chosen as the two variables of this experiment, so the interactions between these two variables and more relevant research are suggested to add into the discussion section, in order to make this manuscript more logical and complete.

The SDS and NaOH protocols are currently the most promising in our research area. Most of the recent articles on decellularization focus on these methods of decellularization by varying the concentrations of these products. We have added a section in the discussion to try to explain our choice to the readers.


3. Line44: A full stop is missing in the last sentence, please add it.

Done.

4. Line 160-166: The operation steps of Protocol 2(D2) are not written in detail.

The detailed protocol is the subject of a specific article currently in redaction.

5. Line 220: It is not stated exactly how many times the experiment was repeated.

This was missing from our article and was also pointed out by the editor. We have therefore added a paragraph to our article and a descriptive table. We hope to have made the information more understandable.

6. Line 387：Is there supposed to be a minus sign in front of 72.7.

Thank you for the remark. We have corrected in the manuscript
