# Supplementary material for: Decellularized vascularized bone grafts as therapeutic solution for bone reconstruction: A mechanical evaluation
Source: PLoS One. 2023 Jan 13;18(1):e0280193. doi: 10.1371/journal.pone.0280193 (PMC9838862; doi:10.1371/journal.pone.0280193)
Supplement: Attachment — Submitted filename: Response to Reviewers.docx [file pone.0280193.s006.docx]

Paris, the 04/11/2022

Dear Editor, Dear Reviewers,

We thank you for having considered our paper entitled: Decellularized vascularized bone grafts as therapeutic solution for bone reconstruction: A mechanical evaluation.

We would like to thank you for your comments and questions that will help us to enhance the quality of our article.

In the following section, we respond to specific comments from reviewers. We also pointed in the article the main changes, that we colored according to one who induced it: Academic editor, Reviewer #1.

We thank you for considering another time this reviewed manuscript.

Academic editor,

1. Figure 2 still has no scale bar. It must be mention on the image and not in the title.

We have changed figure 2 to a more readable figure with a scale bar included

2. All formulations need references unless they were extracted by the authors.

Thank you for pointing out this lack. We have added this to our manuscript.

However, we are not sure what you mean by "formulation".

We understood that

(1) we had to put the references of the products used: ex DNase I from bovine pancreas, Sigma-Aldrich®, Darmstadt, Germany

(2) we have also made a call in the text to the mathematical formulas (in the previous revision) ex L245

3. The discussion is still poor. The authors do not need to find the exact topic! The results could be trendly compared to other similar results. These results are in the references that the authors have used them for a comparison in the literature.

We have tried to support our discussion by adding comparisons with the literature on the subject.

4. Using some sentences about that there is no literature for the discussion is not proper and they must be removed or rewritten in a proper state.

We changed this part when we modified the discussion.

5. Please review your reference list to ensure that it is complete and correct. If you have cited papers that have been retracted, please include the rationale for doing so in the manuscript text, or remove these references and replace them with relevant current references. Any changes to the reference list should be mentioned in the rebuttal letter that accompanies your revised manuscript. If you need to cite a retracted article, indicate the article’s retracted status in the References list and also include a citation and full reference for the retraction notice.

We have checked all our references and have not found any retracted items. However, we have added a reference from Ulklejewski (n°58 in our article) which we found interesting to illustrate our discussion.

**Reviewer #1:**

1. This article proves that the decellularization method affects the mechanical results. However, the description of the decellularization method is vague. A detailed description is needed, such as the duration and frequency of each step, the end point of decellularization. These may also have some influence on the biomechanical properties. It is better to supplement this information.

Thank you for the notification. Decellularization via the H2O2 process is not fully described in our article. We currently do not have the rights to publish this method, which will be published shortly in a full article on the subject. This decellularization method has already been published in 2018 (Van Steenberghe and al, Annals of Vascular Surgery, 2018).

We know that this is an important gap in our paper as the concentrations and infusion times can change our results.

2. Line 193: Fig 2. The results of histologic analysis before decellularization are blurred and unclear. In addition, it would be more convincing if the results of histological obtained by different decellularization methods were compared with those before decellularization.

We have modified this figure with a more readable and clearer one. We have not added an image according to the type of decellularization (SDS vs H2O2) because the differences are too small in photographic level.

3. Line 214: The annotations of Table 1 does not indicate the meaning of X properly.

We modified this part in the manuscript.

4. The discussion of biomechanical differences between the two methods is not sufficient. In addition, Line 544-546, "NaOH and H2O2 cause demineralization of the cortical bone (57)". The presentation does not correspond to the original text of the literature.

Thank you for notifying us of this. We have modified the part concerning this reference and we have expanded the conclusion of this article to make it more precise.
