# Supplementary material for: Decellularized vascularized bone grafts as therapeutic solution for bone reconstruction: A mechanical evaluation
Source: PLoS One. 2023 Jan 13;18(1):e0280193. doi: 10.1371/journal.pone.0280193 (PMC9838862; doi:10.1371/journal.pone.0280193)
Supplement: Attachment — Submitted filename: Response to reviewers.docx [file pone.0280193.s007.docx]

Paris, the 02/12/2022

Dear Editor,

We thank you for having considered our paper entitled: Decellularized vascularized bone grafts as therapeutic solution for bone reconstruction: A mechanical evaluation.

We would like to thank you for your comments and questions that will help us to enhance the quality of our article.

In the following section, we respond to specific comments from the Academic Editor. We also pointed in the article the main changes, that we colored in blue.

We thank you for considering another time this reviewed manuscript.

1. Formulations mean equations! The authors have 4 formulations for stress, strain, etc. All they need references

Sorry for this confusion. We added references to all the equations cited in our paper.

2. The authors mentioned "We currently do not have the rights to publish this method, which will be published shortly in a full article on the subject. This decellularization method has already been published in 2018 (Van Steenberghe and al, Annals of Vascular Surgery, 2018).". All these description should be fully described in the text. Moreover, the future work could be mentioned at the end part of the conclusion

Thank you for this comment. We tried to describe the protocol with as much details as possible in the manuscript and we also added a sentence in the conclusion on the future work.

3. No references could be found for 2022 and 2021! This issue is not acceptable for a scientific article, which references are not updated.

We have updated our references to include the latest articles on the subject.

For this reason, we have added the following articles:

- Parmaksiz M, Elçin AE, Elçin YM. Biomimetic 3D-Bone Tissue Model. Methods Mol Biol. 2021;2273:239‑50.
- *Wüthrich T, Lese I, Haberthür D, Zubler C, Hlushchuk R, Hewer E, et al. Development of Vascularized Nerve Scaffold Using Perfusion-Decellularization and Recellularization ». Materials Science & Engineering C, Materials for Biological Applications. déc 2020;117:111311.*
- Gerli MFM, Guyette JP, Evangelista-Leite D, Ghoshhajra BB, Ott HC. Perfusion decellularization of a human limb: A novel platform for composite tissue engineering and reconstructive surgery. PLoS One. 2018;13(1):e0191497.
- *Kurokawa S, Hashimoto Y, Funamoto S, Murata K, Yamashita A, Yamazaki K, et al. In vivo recellularization of xenogeneic vascular grafts decellularized with high hydrostatic pressure method in a porcine carotid arterial interpose model. PLOS ONE. 22 juill 2021;16(7):e0254160.*
- Fooladi S, Faramarz S, Dabiri S, Kajbafzadeh A, Nematollahi MH, Mehrabani M. An efficient strategy to recellularization of a rat aorta scaffold: an optimized decellularization, detergent removal, and Apelin-13 immobilization. Biomater Res. 22 sept 2022;26(1):46.
- Seiffert N, Tang P, Keshi E, Reutzel-Selke A, Moosburner S, Everwien H, et al. In vitro recellularization of decellularized bovine carotid arteries using human endothelial colony forming cells. J Biol Eng. 21 avr 2021;15(1):15.
- *Wang X, Chan V, Corridon PR. Decellularized blood vessel development: Current state-of-the-art and future directions. Front Bioeng Biotechnol. 2022;10:951644.*
- *Adil A, Xu M, Haykal S. Recellularization of Bioengineered Scaffolds for Vascular Composite Allotransplantation. Front Surg. 25 mai 2022;9:843677.*

4. No separated part could be found for the conclusion! Moreover, this part should be rewritten one by one, in bullets, to show the novelty with quantitative data.

We have added a conclusion with bullet points to our article.

5. The standard should be mentioned for all testing types.

Apart from the indentation tests - to which we added the standard reference - the mechanical tests we performed are not part of any standard procedure because they were applied to non-standardized samples. Therefore, no standardization process could be applied, even if the lab is ISO9001 certified.

6. No quantitative results could be found in the abstract.

We have changed our abstract to include more quantitative results

7. The quality of Figure 7 is not proper.

We have used a new figure. We wish this one to be more suitable.
